# Physicians’ Opinion Regarding Extended Access to Hormonal Contraception in Switzerland

**DOI:** 10.3390/pharmacy9040184

**Published:** 2021-11-12

**Authors:** Tamara Yous, Samuel Allemann, Monika Lutters

**Affiliations:** 1 Department of Medical Sciences, Private University of the Principality of Liechtenstein, 9495 Triesen, Liechtenstein; 2 Pharmaceutical Care Research Group, Department of Pharmaceutical Sciences, University of Basel, 4051 Basel, Switzerland; s.allemann@unibas.ch; 3 Clinical Pharmacy, Cantonal Hospital of Baden, 5404 Baden, Switzerland; monika.lutters@ksb.ch

**Keywords:** pharmaceutical service, pharmacist prescribing, provider, direct pharmacy access, behind the counter, emergency contraception, bridging

## Abstract

(1) **Background**: Access to hormonal contraceptives (HC) strongly differs between countries and varies from over the counter (OTC) to prescription-only availability. This study aimed to identify opinions among physicians in Switzerland regarding extended access to HC. (2) **Methods**: Web-based survey among physicians (gynecologists, general practitioners, and pediatricians) in Switzerland. (3) **Results**: Hundred sixty-three physicians, mainly gynecologists, participated in this survey and 147 (90%) were included for analysis. A total of 68% (*n* = 100) answered that prescription-only status could be extended under certain conditions but physicians were concerned about patients’ safety (97%, *n* = 142). Moreover, there was concern about insufficient patient education on HC (93%, *n* = 136) and that women may forego preventive examinations (80%, *n* = 118). Participants did not support OTC availability (93%, *n* = 136). Pharmacists prescribing (including initiation of HC) revealed controversial results, but a combined access model (initial prescription from physician and follow-up prescriptions by pharmacists) found acceptance in 70% (*n* = 103). (4) **Conclusions**: Participating physicians stated that prescription-only status for HC could be lifted under certain conditions but also some concerns, e.g., patients’ safety or neglection of preventive examinations, were raised. Future research should focus on specific conditions in which extended access to HC could be agreed on.

## 1. Introduction

Over 60 years ago, the first combined oral contraceptive named Enovid^®^ has been approved by the FDA [1]. Nowadays, access to hormonal contraception strongly differs between countries and ranges from prescription-only status to over the counter (OTC) availability. An analysis in 2015 showed that only 47 of 147 countries evaluated required a prescription to obtain hormonal contraceptives (HC) [2]. Kennedy et al. showed in a recent review that women and providers generally supported extended access, meaning access without prescription from a physician [3]. So far, prescription status remained unchanged in the United States of America (USA) but many states allow trained pharmacists to offer contraception service and directly provide HC in community pharmacies [4]. Fifteen states in the US have introduced legislation in 2019 to allow pharmacists prescribing HC [5]. The movement to extended access is not limited to the US. In Canada, pharmacists prescribing and renewing met strong support across the country and some provinces already allow pharmacists to prescribe HC [6]. Midwives certified by the British Columbia College of Nurses and Midwives, are also allowed to prescribe, order, and administer contraceptives [7]. New Zealand has recently reclassified selected HC to allow supply by pharmacists, but first-time users and women aged less than 16 years are excluded [8,9]. In Europe, a study from the United Kingdom (UK) demonstrated that trained community pharmacists provided appropriate oral contraception service and that the pharmacy is a feasible site to provide HC [10]. Recently, progesterone-only pills (POP) have been reclassified in the UK and women can obtain them from pharmacies without prescription [11].

The European Contraception Policy Atlas recommends Switzerland to make self-administered HC available without prescriptions to reduce barriers [12]. Despite this recommendation, opposition to extended access exists and various concerns have been raised [3]. Safety concerns about HC focus mainly on the risk of venous thromboembolism (VTE) associated with combined hormonal contraceptives (CHC). Serious side effects like VTE are rare in women of reproductive age and benefits continue to outweigh their risks in most women [13,14,15,16].

Availability of self-administered HC is not completely new in Switzerland. Pharmacists are embedded in sexual and reproductive health care, as they offer counselling for emergency contraception without physician’s prescription [17]. However, HC still requires a prescription. Although Swiss law allows pharmacists to dispense prescription-only drugs (including HC) in justified cases without a valid prescription, initiation of HC, changing between different methods, substances or dosages are currently not supported. In 2019, the government introduced a new law with the aim to simplify the access of certain medicinal products subject to prescription [18]. Under this new law, prescription-only drugs will be revised and pharmacists providing access to HC could be a new strategy to facilitate access to birth control. In 2020, a survey was conducted among pharmacists in Switzerland in order to gain insight regarding their opinion and interest in extended access to HC [19]. However, no data are available for physicians in Switzerland. The goal of this study was to gain knowledge about physicians’ view on extended access to HC in Switzerland.

## 2. Materials and Methods

We conducted a web-based survey among physicians (GY = gynecologists, GP = general practitioners, PE = pediatricians) between April and May 2021. Recruitment for GY occurred in the newsletter of the Swiss Society for Gynecology and Obstetrics (SGGG). For the recruitment of GP and PE we invited physicians with a registered email address on www.doctor.ch, accessed on 11 May 2021. The Cantonal Ethics Committee of Zurich confirmed that the authorization from the ethics committee is not required for this study.

We calculated sample size with a confidence interval of 95% and a margin of error of 8%. The margin of error is a range that sample survey data are accurate when compared to the population and not identical to the level of significance. The minimal sample size is *n* = 150 physicians, assuming > 10 000 physicians (GY, GP, PE) in Switzerland. The survey was sent to 4839 physicians (2472 GY; 2367 GP/PE), assuming a response rate of about 3–5%.

The questionnaire was administered using the web-based survey tool SoSci Survey (Version 3.2.05-i) [20]. The survey was provided in German and French. Items of interest are based on our previous research among pharmacists [19] and few new items were developed for this survey among physicians and optimized in a multiple-stage process. The questionnaire was tested in pilot trials. As proposed by Kallus, the quality of the translation was verified by back translation [21]. The survey covered demographics and a total of 9 questions with predetermined answer choices. A four-point Likert scale (e.g., No = 0; Rather no than yes = 1; Rather yes than no = 2; Yes = 3), an abstention, as well as options for free-text comments were provided. The questionnaire included the following topics: advantages of extended access to HC, potential barriers (from the concerned women’s point of view), opinion about different prescription models and situations for extended access, patients’ safety, concerns about extended access to HC, and opinions on related statements.

Although the wording “pharmacists prescribing” is not common in Switzerland, we used this expression in the context of extended access to HC through pharmacists. For this survey we defined that birth control service/extended access to HC would include counselling, screening for contraindications, as well as prescribing, dispensing and administering HC. In case of relevant contraindications, a referral to a physician was foreseen.

Inclusion criteria for further analysis was a fully completed questionnaire. Analysis was conducted using SPSS (IBM Corp. Released 2020. IBM SPSS Statistics for Mac, Version 27.0. Armonk, NY, USA) and Microsoft^®^ Office Excel (for Mac, Version 16.50). Data were analyzed using descriptive statistics. For the hypothesis testing, groups were formed according to the hypothesis. For a multi-group comparison of scaled variables, the Kruskal–Wallis Test, and for the comparison of the two groups of scaled variables Mann–Whitney-U Test were performed. Categorical variables were analyzed by Chi-Square Test (χ^2^). In case of more than 20% of cells with expected frequencies < 5, Fisher’s Exact Test was used. In case of significant differences for the overall testing, post-hoc tests (Mann–Whiney-U for scaled variables, χ^2^ for categorical variables) were performed. Bonferroni method was used to adjust significance levels for multiple testing when appropriate. Cramer’s V (V) was used as the effect size for χ^2^ and Cohen’s-d (d) for the two-group-comparison for a continuous characteristic (age).

## 3. Results

### 3.1. Participants’ Characteristics

In total 163 physicians participated in this survey, which corresponds to 3.4% of invited physicians with the assumption that all invitations have reached recipients. A total of 147 questionnaires (90%) met the inclusion criteria and were included for further analysis. With the achieved sample size of 147 a margin of error of 8.08% could be reached. Participants took on average 6 min (SD: 1 min; min-max: 3–9 min) to complete the questionnaire. Participants’ characteristics are displayed in Table 1, showing that participants were mainly GY. Most participants worked in urban areas. The vast majority worked in a doctor’s office (63%, *n* = 93; data not shown) whereas 37% were employed in hospitals (*n* = 54; data not shown). Most participants took the survey in German (84%, *n* = 123; data not shown).

### 3.2. Potential Barriers to Access Hormonal Contraception

**Figure 1:**
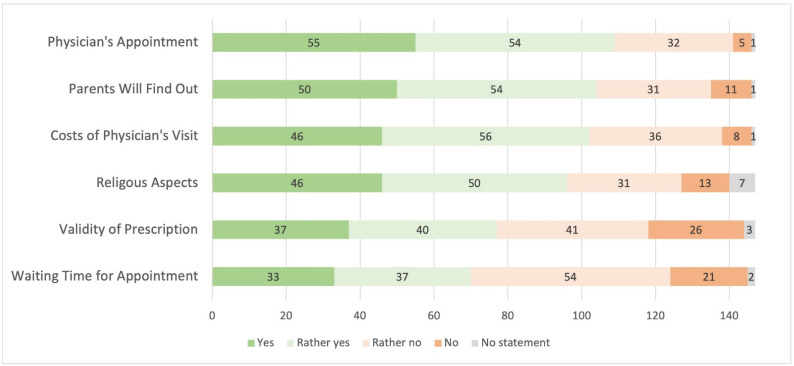
Potential barriers to access hormonal contraception from the concerned women’s point of view (*n* = 147).

Physicians were asked to answer this question from the concerned women’s point of view (Figure 1). A total of 74% (yes or rather yes: *n* = 109) answered that the necessity for a physician’s appointment can display a barrier. Nearly half of participants answered that the waiting time for an appointment can display a barrier to access HC (yes or rather yes: 48%, *n* = 70). Only 3% added a comment and 3 out of 5 participants mentioned “no reimbursement of HC” as additional barrier.

### 3.3. Advantages of Extended Access to Hormonal Contraception

**Figure 2:**
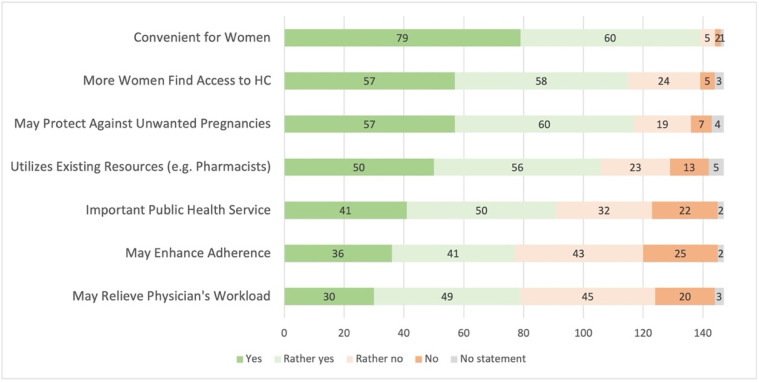
Advantages of extended access to hormonal contraception (*n* = 147; HC = hormonal contraceptives).

Physicians’ opinion regarding advantages are summarized in Figure 2. Significantly more physicians working in urban areas considered it an advantage and answered that extended access to HC may enhance adherence (58% support in urban areas (*n* = 66) vs. 34% in rural areas (*n* = 11), χ^2^ (1) = 5.78, *p* = 0.026, V = 0.20). Furthermore, more hospital-based physicians selected that utilizing existing resources (like pharmacists) is an advantage (89% agreement of hospital-based physicians (*n* = 48) vs. 66% not working in hospitals (*n* = 58), χ^2^ (1) = 9.34, *p* = 0.003, V = 0.26).

### 3.4. Different Access Models and Situations for Extended Access to Hormonal Contraception

**Figure 3:**
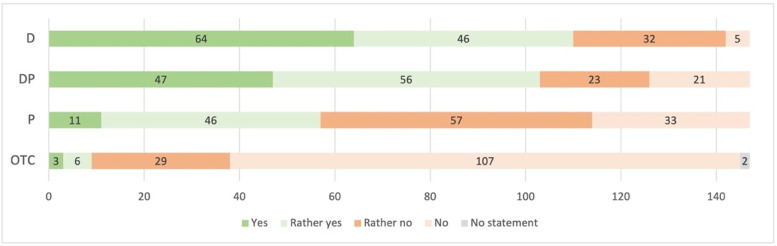
Access models to hormonal contraception (*n* = 147; D = prescriptions solely issued by physicians (doctors); DP = initial prescription from physician, follow-up by pharmacists; P = initial prescriptions by physicians or pharmacists; OTC = over the counter).

Four potential access models were displayed, and results are summarized in Figure 3. There was near-unanimity regarding potential OTC access to HC, as a total of 136 participants voted against it (no or rather no: 93%). Controversial findings were identified for initial prescriptions issued by pharmacists or physicians (yes or rather yes: *n* = 57, 39%). The combined access model, where physicians initiate HC and pharmacists issue follow-up prescriptions, revealed comparable agreement as the model of solely physicians issuing prescriptions (yes or rather yes: *n* = 103, 70%; respectively *n* = 110, 75%).

Furthermore, we asked participants in which situations they would support extended access through pharmacists. According to participating physicians, pharmacists should not be allowed to prescribe HC for first-time users (no or rather no: 86%, *n* = 126). Furthermore, switching between different preparations did not find wide acceptance (no or rather no: 73%, *n* = 107), but significant more physicians from urban areas supported this (32% acceptance in urban areas (*n* = 36) vs. 6% acceptance in rural areas (*n* = 2), χ^2^ (1) = 7.96, *p* = 0.005, V = 0.23). A total of 80% agreed on pharmacists issuing follow-up prescriptions for the same preparation (yes or rather yes: *n* = 117). Younger physicians were more likely to support follow-up prescriptions from pharmacists (47 ± 13 vs. 57 ± 12 years (mean ± SD); *p* < 0.001, d = 0.77).

### 3.5. Concerns about Extended Access to Hormonal Contraception

#### 3.5.1. Patients’ Safety

**Figure 4:**
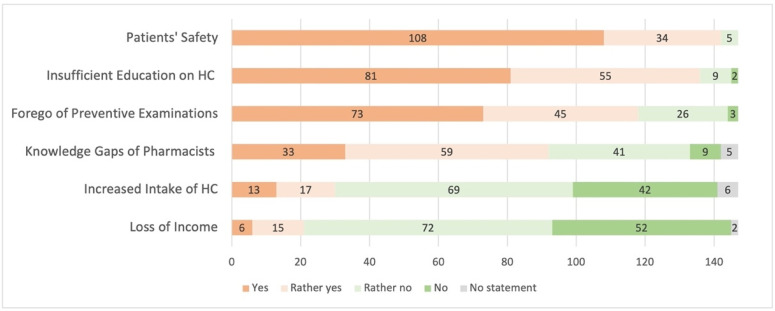
Concerns about extended access to hormonal contraception (*n* = 147; HC = hormonal contraceptives).

Among investigated concerns, patients’ safety was the most common concern (yes or rather yes: 97%, *n* = 142; Figure 4). Due to the different contraindications, opinion on safety was also investigated separately for CHC, POP, and DJ (depot injection) and for different access models. Overall, the same patterns could be observed: participants declared highest safety when HC are prescribed by physicians. More detailed, safety was rated highest in case of physicians’ prescriptions for CHC, POP, and DJ (high or rather high safety: 99% for CHC and POP, 97% for DJ). Patients’ safety was rated lower when HC are also initiated by pharmacists (high or rather high safety: 37% for CHC, 57% for POP and 35% for DJ). Overall, patients’ safety for combined access model, involving pharmacists in follow-up prescriptions, was rated in between (high or rather high safety: 79% for CHC, 81% for POP and 63% for DJ). Younger physicians were more likely to rate patients’ safety higher for POP initiated by pharmacists (45 ± 12 vs. 54 ± 13 years (mean ± SD); *p* < 0.001, d = 0.65). The same applies for DJ (44 ± 11 vs. 51 ± 13 years (mean ± SD); *p* = 0.002, d = 0.56). Overall, more GY reported that POP can be safely prescribed by pharmacists (66% approval by GY (*n* = 68) vs. 37% approval by other physicians (*n* = 15), χ^2^ (1) = 10.41, *p* = 0.002, V = 0.27).

#### 3.5.2. Other Concerns

The concerns examined are summarized in Figure 4. For example, physicians were highly concerned about insufficient patient education (yes or rather yes: 93%, *n* = 136). Another major concern was that women may forgo preventive examination (yes or rather yes: 80%, *n* = 118). Virtually no concerns could be identified for “increased intake of HC” (no or rather no: 76%, *n* = 111) and “loss of a relevant source of income” (no or rather no: 84%, *n* = 124). A total of 9 participants (6%) used the free-text field to address further concerns, but only few of them were not already embedded in the survey. Concerns about less personalized prescriptions or that pharmacists do not have enough time to give all information that is needed for HC were mentioned.

### 3.6. Opinion about Various Statements

More than two-thirds (yes or rather yes: 68%, *n* = 100) answered that prescription-only status could be extended under certain conditions. A total of 80% (yes or rather yes: *n* = 118) agreed that relevant contraindications can be identified with evidence-based tools and physicians working in hospitals were more likely to support this statement (93% support from physicians working in hospitals (*n* = 50) vs. 76% not working in hospitals (*n* = 68), χ^2^ (1) = 6.62, *p* = 0.013, V = 0.21). Nearly half of participants (yes or rather yes: 44%, *n* = 64) indicated that a gynecologic exam is usually required for initiating HC, but significantly more GY answered that such an examination is usually not required to initiate HC (62% GY (*n* = 65) vs. 43% other physicians (*n* = 18), χ^2^ (1) = 4.43, *p* = 0.043, V = 0.17). Fifty-six percent (yes or rather yes: *n* = 82) and significantly more hospital-based physicians (74% (*n* = 39) vs. 48% (*n* = 43), χ^2^ (1) = 9.08, *p* = 0.003, V = 0.25) answered that with extended access to HC competences of other professionals could be better utilized. About half of the participants (yes or rather yes: 52%, *n* = 77) agreed that HC can also be prescribed by other trained staff, such as pharmacists. A vast majority (yes or rather yes: 88%, *n* = 130) supported that the capability of judgement should be considered and about 25% (yes or rather yes: *n* = 36) would support the introduction of a minimum age for extended access to HC. A total of 7 participants (5%) used the free-text field and three participants mentioned that also other professionals could be involved in extended access to HC, e.g., midwives, nursing professionals, or pharmacy assistants.

## 4. Discussion

To our knowledge, this was the first survey among physicians in Switzerland regarding their opinion on extended access to HC. Most participating physicians answered that prescription-only status for HC could be extended under certain conditions.

### 4.1. Practical Implications

Participating physicians raised concerns, e.g., patients’ safety, especially when pharmacists would initiate CHC or DJ. Among other things, this opinion could be explained by the lack of knowledge about the pharmaceutical education and training, as well as about opportunities for pharmaceutical services in pharmacies. Unsurprisingly, there was less concern about patients’ safety for POP, especially among younger physicians. This finding can be explained due to the different safety profile of POP and is in line with recent research in the UK, where respondents were largely supportive of pharmacy-led provision of HC and initiation of POP was most strongly supported [22]. Our survey revealed a clear refusal of OTC access to HC, which corresponds to the view among pharmacists in Switzerland [19]. This attitude is also in agreement with the “conservative attitude” among German pharmacists to a possible OTC switch of HC in Germany, whereas patients and physicians were partly open to it, especially younger physicians (<50 years) [23]. Our study found some significant differences in physicians’ age with medium effect size, indicating that younger physicians might be more open to a switch of HC and/or the involvement of pharmacists in new services. In contrast to OTC accessibility, involved pharmacists insure the patient-healthcare-interaction prior to prescription. However, the American College of Clinical Pharmacy and the American College of Obstetricians and Gynecologists (ACOG) assessed HC to be sufficiently safe to be released from prescription-only status and the ACOG supported OTC-availability of HC [24,25,26].

In the UK, a majority of delegates at national and regional sexual and reproductive health services were supportive of pharmacists providing HC [22] and recently the first POP has been reclassified and is available from pharmacies without a prescription [11]. This is an important first step in the direction of extended access and women empowerment. But having only POP available in pharmacies impedes personalized birth control. POP should not be chosen because it is the only hormonal method available without prescription. Furthermore, serious side effects among women in reproductive age are rare, but some women may be at risk of thromboembolism associated with CHC. However, it is also a fact that pregnancy raises the risk to an even larger degree [16,24,27]. In addition, no pharmaceutical active substance is completely without risk of harm. The World Health Organization (WHO) provides “Medical eligibility criteria for contraceptive use” (MEC), containing a guidance and recommendations on the safety of various contraceptive methods and how to use them [15]. It has been demonstrated that relevant contraindications to HC can be identified with existing tools, concluding that pharmacists can efficiently screen women for the safe use of HC and are able to select appropriate products [28]. In addition, women have also been shown to accurately self-screen for contraindications to HC [29,30,31]. Other research revealed no difference in absolute contraindication between OTC access and family planning clinics (category 4 contraindications according to MEC from WHO) [32]. Therefore, evidence indicates that pharmacists can identify women with relevant contraindications. 

The identification of women at risk can be standardized and facilitated with appropriate supporting materials, e.g., checklists and/or algorithm. In our survey, a majority agreed that relevant contraindications can be detected with evidence-based tools. For example, the California State Board of Pharmacy already provides such a questionnaire, albeit for women in order to complete it before visiting the pharmacy [33]. In Switzerland, the SGGG published a similar checklist for physicians who prescribe CHC [34]. In addition, telehealth has grown and these programs utilize questionnaires to determine patient’s eligibility to HC similar to that used in pharmacies [5,35]. According to the Center for Disease Control and Prevention, blood pressure measurement is the only test needed for a safe use of CHC [36] and this measurement is already routinely offered in Swiss pharmacies.

Most participants did not support initial prescriptions from pharmacists, probably due to concerns about ineligible women using CHC, but they agreed on follow-up prescriptions issued by pharmacists. As relevant contraindications may also develop over time, an initial physician’s prescription may therefore not necessarily increase safety but underlines the importance of accurately trained providers. Surprisingly, a large proportion answered that gynecological examinations are usually required to initiate HC. It is undisputed that pelvic and breast examinations, screening for cervical neoplasia and sexually transmitted infections are important, but they do not provide information necessary for identifying women who should avoid certain HC or need further evaluation [37]. We found significantly less GY considering such examinations necessary in order to initiate HC. This might indicate that other disciplines may overestimate examinations required to prescribe HC. Nevertheless, clinical examinations are commonly accepted practices before initiating birth control, but such requirements may reduce access to HC and are not required to obtain HC [38]. Participants were also concerned that women may forgo their preventive examinations, but evidence exists that extended access to HC are not steering patients away from preventive care. Landau et al. showed that women not using HC obtained a gynecological preventive examination in the past one or two years [39]. Furthermore, data from California and Oregon showed that nearly 90% of women obtained HC in pharmacies had visited their primary care provider within the last year [40]. Moreover, pharmacists can encourage women to obtain preventive care. Nonetheless, authorizing pharmacists to prescribe HC would be a major change in the health care system with the need for clear referral pathways.

We found physicians being concerned about unknowledgeable pharmacists and they doubt that pharmacists would have enough time for counseling on HC. In contrast to this, the majority of participating pharmacists in our previous survey were not concerned about the lack of time [19]. In addition, Parsons et al. showed that trained community pharmacists provide appropriate contraception service and that pharmacies are reasonable sites to provide HC [10]. On the other hand, a study in the United Arab Emirates revealed that pharmacists provided only suboptimal counseling and the authors discussed that they were probably inadequately trained [41]. Pharmacists’ training in Switzerland consists of a 3-year bachelor and a 2-year master’s degree and a subsequent specialist degree enables to work independently in community pharmacies. A specialized training should be required in order to provide appropriate and safe contraception service. Our previous study showed that participating pharmacists were highly interested in providing contraception services and motivated to complete a special training [19]. This might be explained by the fact, that we found a high proportion of pharmacists regularly challenged with situations where no valid prescription for HC is available and have to help out on a timely basis. Nieuwinckel et al. found Flemish pharmacists to be in a similar situation and concluded that *“this practice anticipates what many health care professionals already suggested or could agree with: extending a prescription to the pharmacists”* [42]. Since pharmacists in Switzerland already provide emergency contraception and this service was recently found to be appropriate [43], as well as considering the aim of the government to simplify access to certain prescription-only drugs, extended access to HC should be discussed. Future research should focus on specific conditions in which extended access to HC could be agreed on. We recommend developing tools like checklists, algorithms, and/or guidelines with clear referral pathways, ensuring evidence-based practices and utmost safety. In order to extend access to HC, mandatory training for pharmacists should be introduced.

### 4.2. Strengths and Limitations

Our study has various strengths. For the first time, we provide data about the opinion of GY, GP, and PE to extended access to HC in Switzerland. Most participants were GY, which was our main target group because prescriptions for HC in Switzerland are predominantly issued by GY. The questionnaire is based on previous research among pharmacists in Switzerland [19]. Moreover, the questionnaire was provided in two languages, both French and German, using state-of-the-art translation methodology [21]. Another important strength is, that the study was not financially supported by interest groups.

The main limitation of our study was the relatively high margin of error due to the small sample size, but overall, our response rate was comparable to similar research among physicians [44]. We expected only a small number of invited physicians to participate in the survey. First, the discussion about extended access to HC beyond emergency contraception is quite new in Switzerland and probably did not gain much attention. Second, we assumed that physicians are not interested in expanding birth control service. Third, the SGGG was the only specialty society that distributed the survey, underlining the importance of their involvement and this might explain why only few GP and PE have participated. Other specialty societies were not interested in distributing the survey and wanted to focus on other and eventually more relevant topics for their daily business. Furthermore, the survey was conducted during the COVID-19 pandemic when the health care system in Switzerland was burdened, contributing to the low participation. In addition, potential confounding influences on results cannot be ruled out, e.g., physicians resisting pharmaceutical services and therefore either not participating or claiming concerns about pharmacists prescribing HC. Mitchell et al. recently discussed various arguments from opponents to pharmacist contraception service and showed that opposition arguments are often not evidence-based [5].

## 5. Conclusions

Our survey provides insight into physicians’ view on extended access to HC in Switzerland. Participating physicians stated that prescription-only status for HC could be lifted under certain conditions. Together with our previous research among pharmacists [19], these findings can inform policy-makers and stakeholders and form the foundation for further discussions. However, this survey was not designed to discuss exact conditions for extended access to HC and these questions should be addressed in further research.

## Figures and Tables

**Table 1:** Participants’ characteristics.

Age (Years)	*n* (%)
<30	11 (7%)
30–39	32 (22%)
40–49	28 (19%)
50–59	41 (28%)
≥60	35 (24%)
Average Age (SD)	49 (13)
Median (min–max)	50 (26–79)
**Gender**	***n* (%)**
Female	97 (66%)
Male	50 (34%)
**Specialization**	***n* (%)**
GY	105 (72%)
GP	27 (18%)
PE	10 (7%)
Other	5 (3%)
**Location**	***n* (%)**
Countryside	32 (22%)
Urban	115 (78%)

*n* = 147; GY = gynecologists, GP = general practitioners, PE = pediatricians.
